# A multi-centre case series of alectinib-related erythrocyte membrane changes and associated haemolysis

**DOI:** 10.1007/s12308-020-00427-3

**Published:** 2021-01-07

**Authors:** Veena Gullapalli, Wen Xu, Craig R. Lewis, Antoinette Anazodo, Giselle Kidson Gerber

**Affiliations:** 1 Prince of Wales Clinical School, University of New South Wales, Sydney, NSW Australia; 2 Princess Alexandra Hospital, Brisbane, Australia; 3 The University of Queensland, Brisbane, Australia; 4 Queensland University of Technology, Brisbane, Australia; 5 Department of Medical Oncology, Prince of Wales Hospital, Sydney, Australia; 6 School of Women’s and Children’s, University of New South Wales, Sydney, Australia; 7 Nelune Comprehensive Cancer Centre, Prince of Wales Hospital, Randwick, Sydney, Australia; 8 Kids Cancer Centre, Sydney Children’s Hospital, Randwick, Sydney, Australia; 9 Department of Haematology, New South Wales Health Pathology, Randwick, Sydney, NSW Australia; 10 Department of Haematology, Prince of Wales Hospital, Randwick, Sydney, Australia; 11 University of New South Wales, NSW Kensington, Australia

**Keywords:** Alectinib, Acanthocytes, Erythrocyte membrane, Haemolysis, Anaplastic lymphoma kinase inhibitor

## Abstract

Alectinib is an orally bioavailable anaplastic lymphoma kinase (ALK) inhibitor indicated for *ALK* mutated non-small cell lung cancer (NSCLC). This case series documents the development of significant erythrocyte membrane changes associated with alectinib use in six patients. Morphological findings found on blood film examination include moderate-marked acanthocytes, spheroacanthocytes, and one case demonstrated moderate schistocytes. Two patients in this multi-centre case series developed grade 1 anaemia, and four patients developed grade 2 anaemia. Two patients suffered significant non-immune-related haemolysis. One patient had a co-existing β thalassaemia trait and required treatment cessation due to severe haemolysis. Low-grade anaemia was seen in 22% of patients using alectinib in the ALEX trial and 5% developed ≥ grade 3 anaemia. Alterations in erythrocyte morphology and membrane structure have not been reported in the safety data and clinical trials to date. Drug-induced acanthocytosis is a rare phenomenon and has previously been reported with high-dose prostaglandin administration only. This case series highlights this important laboratory finding with alectinib use and associated clinical sequelae. Alectinib-associated acanthocytosis is likely to be more prevalent than previously recognised. We also highlight the need for vigilance in haematopathology departments for unexpected laboratory findings associated with novel therapies. These findings can be detected in the post-marketing surveillance phase and may have serious clinical implications for patients.

## Introduction

We have observed significant changes in erythrocyte morphology on peripheral blood film examination of six patients treated with alectinib. Clinical sequelae ranged from asymptomatic anaemia to severe haemolysis.

Alectinib is an orally bioavailable anaplastic lymphoma kinase (ALK) tyrosine kinase inhibitor indicated for non-small cell lung cancer (NSCLC) demonstrating the *EML4-ALK* gene rearrangement [1]. It has also been used in malignancies which demonstrate ALK fusion protein expression including anaplastic large cell lymphoma, neuroblastoma, and inflammatory myofibroblastic tumour [2]. Although in vivo animal studies during the pre-clinical phase reported mild haemolysis [3], to the authors’ knowledge, there are no reports of altered erythrocyte morphology in published trial data. In this multi-centre case series, we report six cases with new onset significant acanthocytosis and sphero-acanthocytosis on peripheral blood film examination.

## Cases reports

**Fig. 1:**
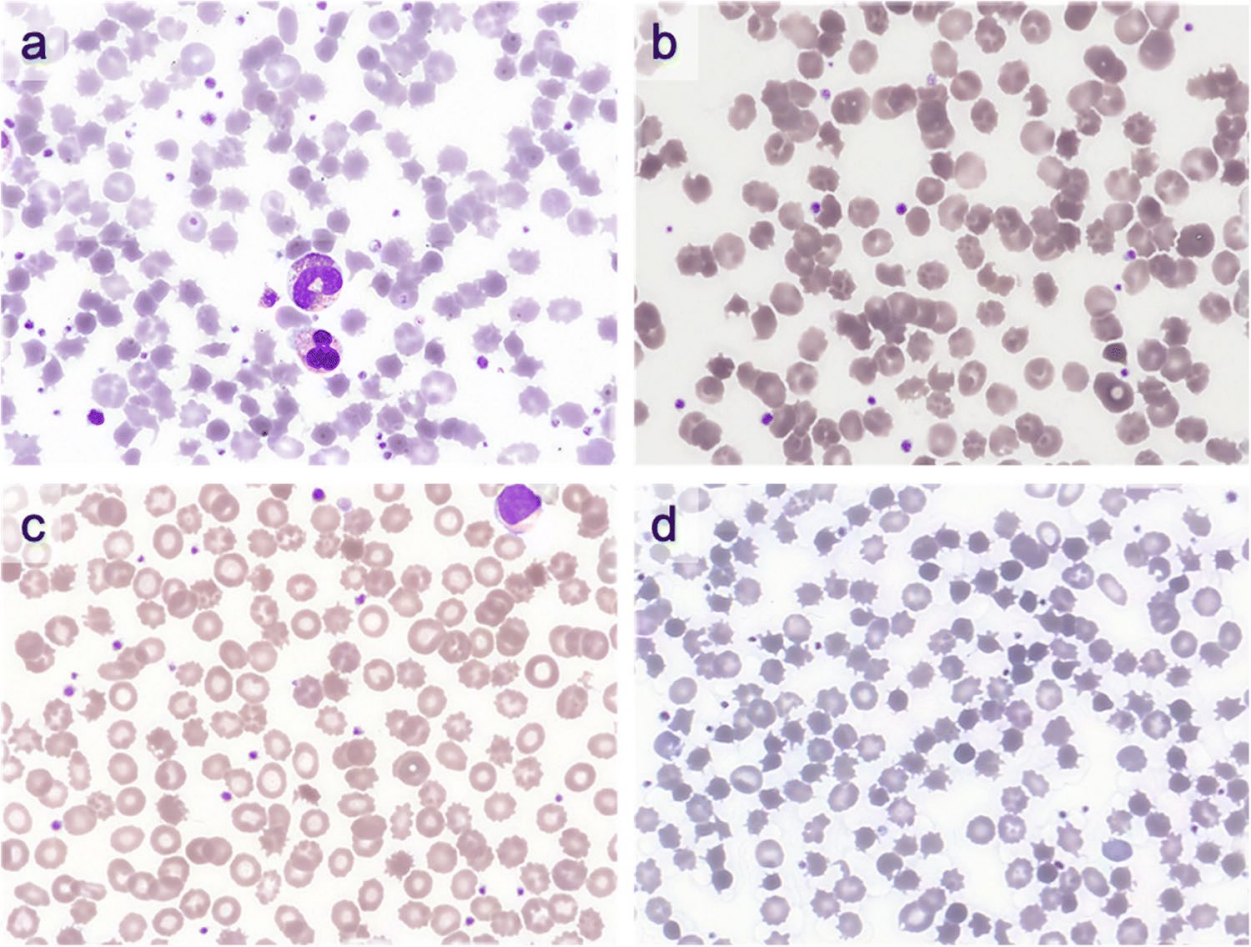
**a** Peripheral blood film (PBF) from blood tests three months after initiation of alectinib in patient 1. It demonstrates a normocytic anaemia with severe acanthocytosis, left shift with neutrophilia, and hypereosinophilia, *× 100 Wright Geimsa*. **b** PBF of patient 2 24 months after starting alectinib demonstrating moderate to severe acanthocytosis, *× 100 Wright Geimsa*. **c** PBF of patient 3 two months of alectinib treatment demonstrating a microcytic hypochromic anaemia and moderate acanthocytosis, *× 100 Wright Geimsa*. **d** PBF of patient 6 three months after alectinib was initiated demonstrating severe acanthocytosis and spherocanthocytes with reticulocytosis, *× 100 Wright Geimsa*

The index case was a 79-year-old female who presented with a malignant pleural effusion secondary to metastatic ALK-positive NSCLC. She commenced ceritinib, however gastrointestinal toxicity and hepatic transaminitis resulted in treatment change to alectinib. Three months after commencement of alectinib, she was admitted to hospital for recurrence of the malignant pleural effusion. There was a new normocytic normochromic anaemia with haemoglobin 100 g/L and MCV 88 fL. The blood film demonstrated acanthocytes, spherocytes, left shift with neutrophilia, and hypereosinophilia (Fig. 1a). Renal function, liver function tests, and electrolyte profile were within normal limits. Lipid profile or thyroid function tests were not available. The patient subsequently died from progressive lung cancer.

Patient 2 was a 66-year-old male incidentally found to have moderate acanthocytes and mild spherocytes on a blood film examination (Fig. 1b) following commencement of alectinib for relapsed ALK-positive lung adenocarcinoma in mid-2017. Haematological indices prior to initiation of alectinib were within normal parameters. Blood film prior to initiating therapy was unavailable. During therapy with alectinib, the patient developed hypothyroidism and was started on thyroxine 100 mcg daily. Haematological indices performed at the time of the blood film demonstrated a normocytic anaemia with haemoglobin of 107 g/L, MCV 87 fL, and RDW 42 fL. Lactate dehydrogenase (LDH) level was mildly elevated at 266 U/L (150–250 U/L), and haptoglobin was 0.8 g/L (0.3–2.0 g/L). The patient’s renal function, liver function tests, electrolyte profile, and lipid profile were normal.

Patient 3 is a 14-year-old female who was diagnosed with metastatic ALK-positive lung adenocarcinoma and commenced treatment with alectinib. She was also taking trimethoprim-sulfamethoxazole for *Pneumocystis jirovecii* prophylaxis. Prior to initiation of alectinib, haemoglobin was 107 g/L, MCV 71 fL, platelet count 394 × 10^9^/L, and WCC 7 × 10^9^/L. Iron studies demonstrated a mild iron deficiency with ferritin 49 μ/L and transferrin saturation of 4%. A haemoglobin electrophoretogram was normal. Blood tests undertaken two months after initiating alectinib demonstrated a persistent mild microcytic anaemia with haemoglobin of 96 g/L and MCV 71 fL. The blood film (Fig. 1c) showed marked microcytosis and moderate acanthocytes. Thyroid function tests were unremarkable. The patient remains in remission from the lung adenocarcinoma.

**Fig. 2:**
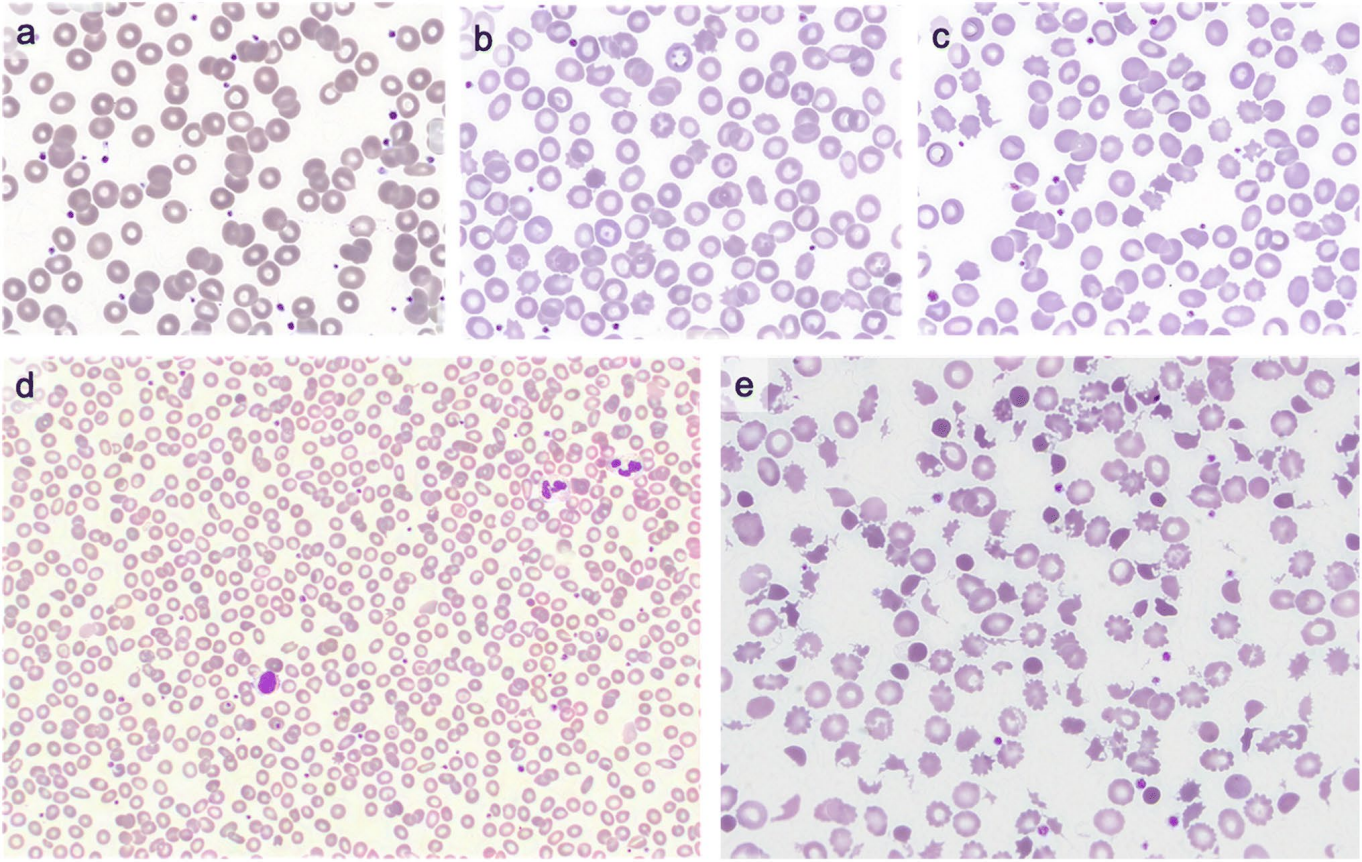
**a** PBF of patient four prior to initiating alectinib demonstrating normocytic normochromic cells, *× 100 Wright Geimsa*. **b** PBF day 50 post-alectinib demonstrating occasional acanthocytes in patient 4, × *100 Wright Geimsa*. **c** PBF day 127 post-alectinib demonstrating moderate acanthocytes in patient 4, × *100 Wright Geimsa*. **d** PBF of patient 5 prior to the initiation of alectinib which demonstrates microcytic anaemia, basophilic stippling, mild anisopoikilocytosis. and occasional target cell consistent with β thalassemia trait, *× 40 Wright Geimsa*. **e** PBF after one month of therapy for patient 5 showing marked fragmentation and marked anisopoikilocytosis, with irregularly contracted cells, acanthocytes, microspherocytes, and polychromasia. There is background microcytosis and basophilic stippling, consistent with known β thalassaemia trait, *× 100 Wright Geimsa*

Patient 4 is a 30-year-old female who was diagnosed with a retroperitoneal primary neuroblastoma at age 19. Prior treatments included chemotherapy, surgery, radiotherapy, and cis-retinoic acid. Recurrence four years later was treated with surgery and radiotherapy. Following a further recurrence, molecular analysis identified ALK fusion protein and the patient commenced alectinib. The initial blood film demonstrated a mild normocytic normochromic anaemia with normal red cell morphology (Fig. 2a). Blood films generated on D14, D50, and D127 of therapy (Fig. 2b & 2c) demonstrated increasing numbers of acanthocytes and there was a mild improvement in haemoglobin (Table 1). Renal function, liver function, electrolyte profile, thyroid function, and lipid profile remained within normal limits.

**Table 1:** Haematological indices and biochemistry during different time points of alectinib therapy

	Case 1		Case 2		Case 3				Case 4				Case 5				Case 6					
Age (years)	79		66		14				30				66				77					
Gender	Female		Male		Female				Female				Male				Female					
Days post starting alectinib	0	98	0	651	0	54	84	1014	0	22	128	778	0	28	21 days post-alectinib cessation	42 days post-alectinib cessation	0	56	84	14 days post-alectinib cessation	42 days post-alectinib rechallenge at 450 mg bd	84 days post-alectinib rechallenge at 450 mg
Haemoglobin 115–165 g/L	116	91	131	107	107	104	96	113	110	107	116	104	111	81	80	100	133	110	104	94	98	95
MCV 89-98 fL	85	90	88	87	72	72	71	84	84	82	93	95	63	57	66	66	86	87	88	88	85	85
MCHC 310-360 g/L	331	324	335	357	324	313	325	336	322	323	326											
RDW 38-48 fL	45	63	40	42	36	48	52	49	47	51	51											
Platelets 150-450 × 10^9^/L	446	659	191	237	383	370	284	250	487	395	334	165	210	253	247	336	339	327	405	712	507	594
White cell count 3.5–11 × 10^9^	6	20	5.6	5.6	5.4	6	4.9	10.1	7.9	7	5	3.7	7.4	7.2	6.1	5.5	8.7	8.3	12.1	12.2	7.3	10.4
Bilirubin < 19umol/L	12	10	6	21	4	17	17	26	5	5	6	4	14	42		16	30	69	105	26	33	49
ALP 30–110 U/L	974	150	101	65	130	200	197	139	110	110	130	186	45	82		51	53	98	124	83	97	107
GGT < 35 U/L	810	124	248	20	19	13	10	9	23	23	12	22	18	10		17	31	19	22	29	27	22
ALT 10–35 U/L	223	13	151	21	15	48	29	21	22	22	22	28	17	104		17	32	24	23	18	20	16
AST 10–35 U/L	97	20	71	24	22	55	35	36	43	43	42	39	19	131		22	18	21	19	16	18	22
LDH 150–250 U/L				266	190			335				233	229	836		248	247	292	317	293	306	309
Haptoglobin 0.3–2.0 g/L				0.8				0.9				1.4		0.05	0.57	1.38			0.97	1.53	0.26	0.02
Reticulocyte count 30–140 × 10^9^/L								147				436		137	183	96			100	161	102	124
Ferritin 30–150 μg/L					49			48														
Transferrin saturation 16–51%					4			11														
Direct antiglobulin test								Negative						Negative				Negative				
Haemoglobin electrophoretogram								Normal														

Patient 5 is a 66-year-old male treated for stage II NSCLC in 2014 with a lobectomy, followed by adjuvant chemotherapy with cisplatin and vinorelbine. Past medical history included localised prostate cancer which remains under surveillance and known β thalassaemia trait. In 2015, he developed multiple cerebral metastases and received whole brain radiotherapy. In 2020, he developed recurrent brain metastases. Presence of an ALK fusion gene was confirmed and alectinib was commenced. Baseline haemoglobin was 115 g/L, with an MCV of 62 fL and the film demonstrated microcytosis and basophilic stippling, consistent with known β thalassaemia trait (Fig. 2d). After a month on alectinib, he developed symptomatic anaemia with haemoglobin of 81 g/L. Haemolysis was confirmed with an unconjugated hyperbilirubinaemia to 31 μmol/L, elevated reticulocyte count of 137 × 10^9^/L, elevated LDH of 836 U/L, and low haptoglobin of 0.04 g/L. The direct antiglobulin test (DAT) was negative. Concurrent with this, there was a new grade 3 AST and ALT derangement (Table 1). His platelet count remained in the normal range and an ADAMTS13 level was normal at 0.67 IU/mL (RR 0.40 to 1.30 IU/mL). The blood film demonstrated marked acanthocytosis, fragmentation, microspherocytes, and polychromasia. Alectinib was ceased with a plan to rechallenge with an alternative ALK inhibitor upon disease progression, which has not yet occurred. Post-alectinib cessation, follow-up blood films demonstrated persistent morphological changes, but normalisation of haemolytic parameters, with recovery of haemoglobin to 100 g/L by 6 weeks (see Table 1).

Patient 6 is a 77-year-old female diagnosed with ALK-positive NSCLC with nodal and pleural metastases. Comorbidities included obstructive airways disease and hypothyroidism for which she was taking thyroxine 50 mcg daily. Three months post-initiation of alectinib 600 mg BD, her haemoglobin had decreased from a baseline of 133 g/L to 104 g/L with evidence of DAT-negative haemolysis (Table 1). The blood film showed marked spheroacanthocytes, with minimal red cell fragmentation (Fig. 1d). Alectinib was withheld with improvement of anaemia and normalisation of haemolysis markers after two weeks. She was rechallenged at the modified dose of alectinib at 450 mg BD, but after 12 weeks, there was recurrence of haemolysis with hyperbilirubinaemia to 49 μmol/L, reticulocyte count of 124 × 10^9^, and a decreased haptoglobin to 0.02 g/L. Alectinib was ceased permanently, and brigatinib, an alternative ALK inhibitor, commenced. Four weeks after cessation of alectinib and initiation of brigatinib, haematological indices indicated complete resolution of haemolysis, confirming this was likely an alectinib-specific effect.

## Discussion

We have reported six cases of significant alterations in erythrocyte morphology secondary to alectinib. Case 4 demonstrated this transformation of normal erythrocytes to those with spiculated membranes after initiation of alectinib. Patients predominantly demonstrated mild anaemia, however two patients developed significant DAT-negative haemolysis and one demonstrated severe erythrocyte fragmentation. In these cases, there was significant improvement in haemolytic markers after alectinib cessation. Significant haemolysis recurred after re-initiation of alectinib at a reduced dose in case 6. This indicates a possible effect of alectinib on the metabolism and membrane integrity of erythrocytes. These changes can be seen soon after initiating therapy. It remains unclear whether this is a dose-dependent effect.

Low-grade anaemia is a common toxicity seen with alectinib and was reported in 14–22% of patients in studies [1, 4]. Although the majority of alectinib-induced anaemia is grade 1 or 2 severity, clinical trials report that 1–5% of the patients experience grade 3 or worse anaemia [1, 4, 5]. The recent safety and efficacy update from phase 3 ALEX trial demonstrated that ≥ grade 3 anaemia was more frequent with alectinib when compared to crizotinib [3]. Mild increases in bilirubin are also commonly observed with alectinib, with an overall incidence of 15% in the ALEX trial [1] and 2% experiencing ≥ grade 3 bilirubinaemia. This raises the suspicion of underlying haemolysis. Although animal studies have shown evidence of alectinib-induced abnormal red cell morphology and increase in reticulocytosis [3], to our knowledge, blood films or haemolytic parameters were not routinely monitored in the clinical trials.

Acanthocytes refers to contracted erythrocytes exhibiting multiple membrane projections which contrast with the recognisable discoid structure. This is seen in conditions which alter the ratio sphingomyelin and glycerophospholipids [6] or alter the anchorage of the cytoskeleton network to the erythrocyte membrane [7]. It is more commonly seen in advanced cirrhosis, malnutrition, hypothyroidism, and inherited disorders such as neuroacanthocytosis and hypobetalipoproteinaemia [8]. Drug-induced acanthocytosis is a rare phenomenon. The only reported case was with high-dose prostaglandin, which was also associated with significant haemolysis [9]. ALK tyrosine kinase activates multiple pathways associated with cell proliferation and differentiation, but the mechanism of alectinib-induced membrane changes remains unknown. The haemolysis seen in acanthocytosis syndromes is hypothesised to be secondary to reduced erythrocyte deformability leading to splenic entrapment and macrophage phagocytosis [10]. However, the numerous fragments in case 5 suggested a component of intravascular haemolysis. We hypothesise that the significant haemolysis seen in this specific case may be due to concurrent haemoglobinopathy further impairing erythrocyte deformability needed to pass through the microvasculature of the spleen [11].

To our knowledge, the described red cell changes are unique to alectinib rather than a class effect. We are not aware of acanthocytosis or haemolysis associated with other ALK inhibitors including crizotinib, ceritinib, brigatinib, or lorlatinib. This was confirmed in case 6, whereby the haemolytic changes resolved after four weeks of ceasing alectinib, despite starting brigatinib. We propose that in the situation where clinically significant haemolysis attributable to alectinib occurs, an alternative ALK inhibitor should be considered. In cases of asymptomatic patients with only morphological changes on blood film, it may be reasonable to continue alectinib with close observation of haematological indices and haemolytic parameters. We would also recommend increased vigilance when using this medication in patients with known haemoglobinopathies.

This report highlights the need for both clinicians and laboratory haematologists to be observant for unrecognised off-target effects of novel agents. We recommend routine blood film examinations and regular haemolytic markers to be undertaken in patients receiving alectinib.
